# Modified Pediatric ASPECTS Correlates with Infarct Volume in Childhood Arterial Ischemic Stroke

**DOI:** 10.3389/fneur.2012.00122

**Published:** 2012-07-31

**Authors:** Lauren A. Beslow, Arastoo Vossough, Hisham M. Dahmoush, Sudha Kilaru Kessler, Rebecca Stainman, Christopher G. Favilla, Courtney J. Wusthoff, Sarah Zelonis, Daniel J. Licht, Rebecca N. Ichord, Sabrina E. Smith

**Affiliations:** ^1^Division of Neurology, The Children’s Hospital of Philadelphia, Perelman School of Medicine of the University of PennsylvaniaPhiladelphia, PA, USA; ^2^Division of Neuroradiology, The Children’s Hospital of Philadelphia, Perelman School of Medicine of the University of PennsylvaniaPhiladelphia, PA, USA; ^3^Department of Neurology, The Hospital of the University of Pennsylvania, Perelman School of Medicine of the University of PennsylvaniaPhiladelphia, PA, USA; ^4^Division of Child Neurology, Stanford University School of MedicineStanford, CA, USA

**Keywords:** modified pediatric ASPECTS, arterial ischemic stroke, MRI, infarct volume, childhood, perinatal

## Abstract

**Background and Purpose:** Larger infarct volume as a percent of supratentorial brain volume (SBV) predicts poor outcome and hemorrhagic transformation in childhood arterial ischemic stroke (AIS). In perinatal AIS, higher scores on a modified pediatric version of the Alberta Stroke Program Early CT Score using acute MRI (modASPECTS) predict later seizure occurrence. The objectives were to establish the relationship of modASPECTS to infarct volume in perinatal and childhood AIS and to establish the interrater reliability of the score. **Methods:** We performed a cross sectional study of 31 neonates and 40 children identified from a tertiary care center stroke registry with supratentorial AIS and acute MRI with diffusion weighted imaging (DWI) and T2 axial sequences. Infarct volume was expressed as a percent of SBV using computer-assisted manual segmentation tracings. ModASPECTS was performed on DWI by three independent raters. The modASPECTS were compared among raters and to infarct volume as a percent of SBV. **Results:** ModASPECTS correlated well with infarct volume. Spearman rank correlation coefficients (ρ) for the perinatal and childhood groups were 0.76, *p* < 0.001 and 0.69, *p* < 0.001, respectively. Excluding one perinatal and two childhood subjects with multifocal punctate ischemia without large or medium sized vessel stroke, ρ for the perinatal and childhood groups were 0.87, *p* < 0.001 and 0.80, *p* < 0.001, respectively. The intraclass correlation coefficients for the three raters for the neonates and children were 0.93 [95% confidence interval (CI) 0.89–0.97, *p* < 0.001] and 0.94 (95% CI 0.91–0.97, *p* < 0.001), respectively. **Conclusion:** The modified pediatric ASPECTS on acute MRI can be used to estimate infarct volume as a percent of SBV with a high degree of validity and interrater reliability.

## Introduction

Arterial ischemic stroke (AIS) affects between 1.2 and 13 pediatric patients per 100,000 person-years in developed countries (Giroud et al., 1995; Fullerton et al., 2003, 2007), and up to 85% have residual neurological deficits (Ganesan et al., 2000). Outcome predictors for pediatric AIS are poorly understood, but recently larger infarct volume has been demonstrated to be a predictor of poor functional outcome as well as a predictor of hemorrhagic transformation (HT) in children (Ganesan et al., 1999; Beslow et al., 2011). However, infarct volume in the pediatric population must be expressed as a percent of brain volume to account for the varying brain size in children of different ages, requiring measurement of total brain volume as well as the infarct volume. Automatic segmentation methods are unreliable in children, particularly in neonates and younger children, due to incomplete myelination. Therefore, these measurements are often performed by manual segmentation techniques that are time consuming and require post-image processing that cannot be performed easily or quickly in real time. As a result, important prognostic information cannot be quantified in the acute setting.

A recent study of neonates with perinatal AIS used a modification of the Alberta Stroke Program Early Computed Tomography Score (ASPECTS) on acute MRI for use in children (modASPECTS) to assess semiquantitatively infarct volume (Wusthoff et al., 2011). Higher modASPECTS predicted seizure recurrence at follow-up. However, the relationship of modASPECTS to actual infarct volume and the interrater reliability of modASPECTS have not been assessed. The objectives of this study were to evaluate the relationship of modASPECTS to infarct volume and to evaluate the interrater reliability of the score in newborns and children with acute AIS.

## Materials and Methods

The study was performed with institutional review board approval.

### Study design

This is a cross sectional study comparing modified pediatric ASPECTS on acute MRI to infarct volume as a percent of supratentorial brain volume (SBV) in a prospectively identified cohort of neonates and children with acute supratentorial AIS.

### Study population

Subjects were identified from a single tertiary care center stroke registry. In the current study, perinatal subjects were ≥37 weeks gestational age with acute AIS within the first 28 days of life and presented between January 1, 2004 and October 31, 2009. Childhood subjects were age >28 days to 18 years inclusive presenting with acute AIS between January 2005 and November 2008. All subjects’ infarcts were confirmed by MRI demonstrating restricted diffusion within an arterial vascular territory conforming to the localization of acute neurologic deficit. For inclusion in the study, an axial diffusion weighted image (DWI) with apparent diffusion coefficient (ADC) map and an axial T2 image were required. The perinatal subjects and childhood subjects were previously described in two papers (Beslow et al., 2011; Wusthoff et al., 2011).

### Study procedure

Infarct location was defined by the arterial territory or territories affected. Additionally, watershed infarcts were defined as infarcts in the borderzone between anterior cerebral artery-middle cerebral artery (ACA-MCA), middle cerebral artery-posterior cerebral artery (MCA-PCA), and internal deep MCA borderzone territories. Some children had watershed ischemia in other regions in addition to their defined vascular territory infarcts.

#### Infarct volume

Infarct volume was measured on axial DWI for both perinatal and childhood subjects by Lauren A. Beslow. DWI was chosen since T2-weighted imaging may not demonstrate the full extent of the lesion if acquired soon after stroke onset, and lesions in the neonates are seen less well on T2 due to incomplete myelination and high water content of the brain. ADC maps were used to confirm areas of acute infarction. Previous adult studies have also used DWI to measure acute infarct volume (Lovblad et al., 1997). SBVs were measured on axial T2 MRI for all subjects (Rebecca Stainman, Christopher G. Favilla, Lauren A. Beslow). We chose T2 images rather than DWI for the SBV measurements because T2 images have better anatomic resolution. Infarct volume was expressed as percent of SBV (excluding ventricular volume) to account for varying head size during development, as previously described (Ganesan et al., 1999; Beslow et al., 2011). Volumes were measured by manual segmentation tracing using ITK-SNAP software (www.itksnap.org; Yushkevich et al., 2006). Manual segmentation requires ∼6 h per subject. This does not include the time for clinical images to be transferred to a research database and then to be reformatted for use in the segmentation program.

#### Modified pediatric ASPECTS

**Figure 1:**
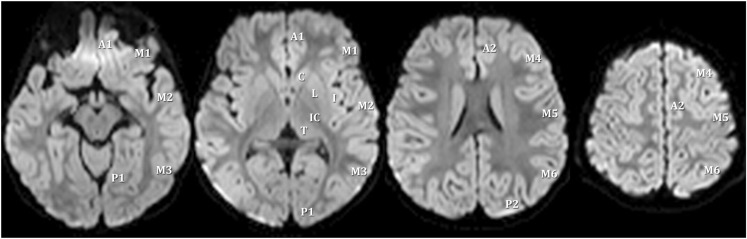
**Scoring areas for the modified pediatric ASPECTS**. A1, proximal anterior cerebral artery territory; P1, inferior portion of posterior cerebral artery territory; M, middle cerebral artery; C, caudate; L, lentiform; I, insula; IC, internal capsule; T, thalamus; P2, superior portion of posterior cerebral artery territory; A2, distal anterior cerebral artery territory.

**Figure 2:**
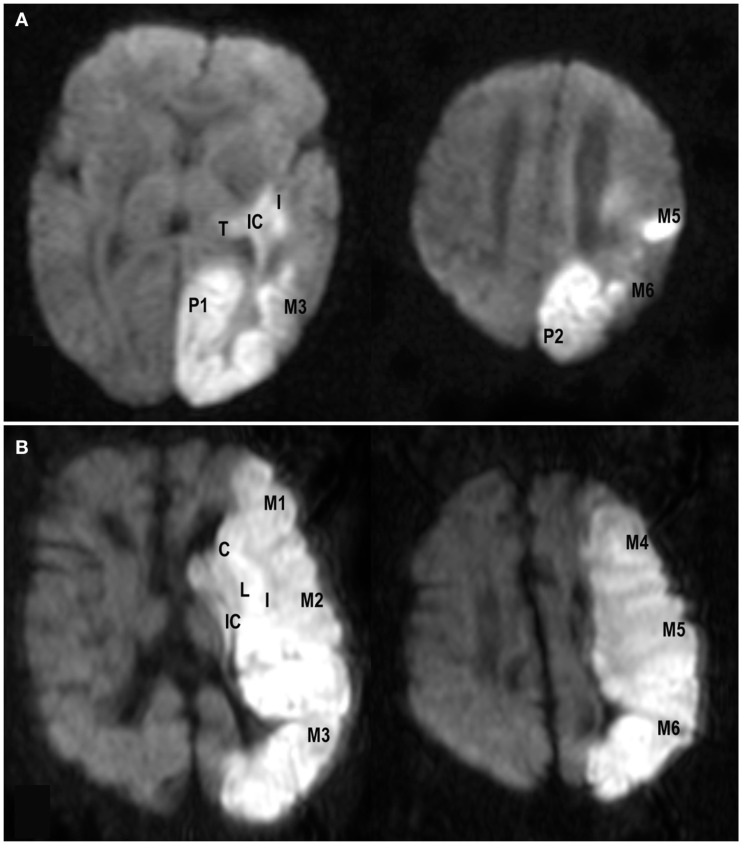
**Examples of axial diffusion weighted images from a neonate (A)** with a modified pediatric ASPECTS of eight (involvement of left internal capsule, insula, M3, M5, M6, P1, P2, and thalamus) and a child **(B)** with a modified pediatric ASPECTS of 10 (involvement of left caudate, lentiform, internal capsule, insula, and middle cerebral artery territories M1–M6).

ASPECTS was designed to provide quick and reliable semiquantitative estimation of early ischemic middle cerebral artery territory infarction on CT in adults (Barber et al., 2000). In the adult ASPECTS, 10 points are assigned for a normal MCA territory and a point is subtracted for each affected MCA region. The adult score has been adapted for use with DWI (Barber et al., 2005; Kimura et al., 2008; Tei et al., 2011). The modified pediatric ASPECTS, as described previously, uses axial DWI to assess supratentorial infarct volume in a semiquantitative way (Wusthoff et al., 2011). Since much of perinatal and childhood stroke affects territories other than the MCA, it was important to expand the pediatric score to include the ACA and PCA territories as well as the thalamus. Additionally, each abnormal region is assigned a point rather than having a point subtracted for the abnormal region as would be done for the adult score. Therefore, for each hemisphere one point is assigned for infarction involving each of the following regions: seven cortical middle cerebral artery regions, two cortical anterior cerebral artery regions, two cortical posterior cerebral artery regions, and four subcortical regions (caudate, lentiform nuclei, internal capsule, and thalamus; Figures 1 and 2). The modified pediatric ASPECTS method takes fewer than 10 min per subject and can be performed at the time the clinical image is obtained without the need for reformatting the images.

For each hemisphere there are 15 regions yielding a maximum of 15 points per hemisphere, and the maximum total modified pediatric ASPECTS score is 30. During scoring, a region was scored as positive even if a small portion of a modified pediatric ASPECTS score region was involved, including watershed areas or punctate foci.

#### Rater training

The raters were a board-certified pediatric neuroradiologist (Arastoo Vossough), a pediatric neuroradiology fellow (Hisham M. Dahmoush), and a pediatric stroke neurologist (Sabrina E. Smith). The three raters performed practice scoring on eight non-study subjects together but performed their ratings on study subjects independently and blinded to the other rater’s scores as well as to the volumetric measurements. Arastoo Vossough and Hisham M. Dahmoush used a clinical image viewing program (iSite, Philips, Netherlands) for viewing the images for their ASPECTS ratings while Sabrina E. Smith used ITK-SNAP for viewing the images for her ratings. Raters were permitted to confirm areas of infarction on DWI for scoring by inspecting the ADC maps.

### Statistical analyses

Descriptive statistics were performed using frequency distributions and proportions for categorical variables and means with standard deviations or medians with interquartile ranges (IQRs) for continuous variables. The perinatal and childhood cohorts were analyzed separately since they represent distinct subgroups of pediatric stroke. Spearman rank correlation coefficient was used as a measure of criterion validity between infarct volume as a percent of SBV and the modified pediatric ASPECTS. A correlation coefficient of >0–0.3 was considered weakly positive, >0.3–0.7 moderately positive, and >0.7–1.0 strongly positive. Pre-specified subanalyses were done with the exclusion of subjects with only multifocal punctate infarcts who were likely to represent a subgroup of subjects in which the modified pediatric ASPECTS was not well correlated with stroke volume. Interrater reliability of the total modified pediatric ASPECTS was determined by the calculation of an intraclass correlation coefficient (ICC) using one-way analysis of variance. An ICC was considered moderate agreement if 0.41–0.60, substantial agreement if 0.61–0.80, and almost perfect (excellent) if 0.81–1.00 (Landis and Koch, 1977). A two-sided probability value of <0.05 was considered statistically significant.

We further evaluated whether modASPECTS can reliably classify subjects as high-risk for poor outcome due to large infarct volume as, previously described, in which infarct volume ≥5% of SBV was predictive of HT and poor outcome (Beslow et al., 2011). Excluding subjects with only multifocal punctate infarcts, the sensitivity and specificity of the modified pediatric ASPECTS for correctly classifying an infarct as large (≥5% of SBV) were calculated in the perinatal and childhood groups, along with area under the receiver operating characteristic curve.

Analyses were conducted using STATA version 11.1 (STATA Corporation, College Station, TX, USA).

## Results

### Patient characteristics

There were 46 potential perinatal subjects with isolated supratentorial stroke previously described in our study evaluating later seizure occurrence after acute perinatal stroke (Wusthoff et al., 2011). Of these 46 subjects, 10 had MRIs performed at other institutions that were non-DICOM format compliant and could not be measured in ITK-SNAP; 2 did not have axial T2 for total SBV measurement; and 3 had DWI that was too degraded for measurement. This left 31 evaluable subjects. There were 49 childhood stroke subjects with isolated supratentorial stroke previously described in our study examining the relationship of infarct volume to HT and to outcome (Beslow et al., 2011). Of these 49 subjects, 4 were excluded because DWI was too degraded for infarct volume measurement, 2 had head CT only, and 3 did not have DWI, leaving 40 evaluable subjects.

There were 71 subjects (31 perinatal, 40 childhood). The median age of the childhood subjects was 3.7 years (IQR; 0.3–12 years). Forty-two subjects (59%) were male. Forty-four (62%) were white (2 Hispanic), 20 (28%) were black, 3 (4%) were mixed race, and 4 (6%) were unknown race or other race. Twenty-eight perinatal subjects (90%) presented with seizures. Thirty-nine childhood subjects (97.5%) presented with focal deficits, and 15 (37.5%) had seizures. HT was present in nine (29%) perinatal subjects and in five (12.5%) childhood subjects. HT was petechial in all perinatal subjects (seven punctate, two confluent), petechial in four childhood subjects (three punctate, one confluent), and small parenchymal in one childhood subject. Presence of HT did not affect the volumetric measurements or the modASPECTS ratings.

### Stroke risk factors

Of the perinatal subjects, 5 (16%) had a cardioembolic stroke, 1 of whom also had an intracranial vasculopathy, 3 (10%) had a thrombophilia, 1 (3%) had *in utero* cocaine exposure, and 22 (71%) had no identified risk factor. Of the childhood subjects, 12 (30%) had cardioembolic stroke, 11 (27.5%) had vasculopathy (4 moyamoya, 4 intracranial vasculopathy, 1 carotid dissection, 1 vertebral dissection, 1 vasculitis), 4 (10%) had tumor-related stroke, 3 (7.5%) had meningitis, 3 (7.5%) had isolated thrombophilia, 2 (5%) had sickle cell anemia, and 5 (12.5%) were cryptogenic.

### Infarct volume and distribution

The median absolute infarct volume on DWI for the perinatal and childhood stroke subgroups was 24.5 ml (IQR 7.8–62.9 ml) and 24.6 ml (IQR 4.5–106.2 ml), respectively. The median infarct volume as a percent of SBV in the perinatal subjects was greater than that for the childhood subjects (6.9%, IQR 2.4–17.2 vs. 2% IQR 0.4–9.8%, *p* = 0.0077, rank sum). Table 1 describes stroke locations among the perinatal and childhood subjects.

**Table 1:** **Stroke distributions**.

Characteristic	Perinatal (*N* = 31)	Childhood (*N* = 40)
Left	14 (45.1%)	20 (50%)
Right	7 (22.6%)	14 (35%)
Bilateral	10 (32.3%)	6 (15%)
Isolated MCA	20 (64.5%; 2 watershed*)	29 (72.5%; 3 watershed*)
Isolated PCA	1 (3.2%)	4 (10%; 1 watershed*)
Isolated ACA	0 (0%)	1 (2.5%)
ACA + MCA	2 (6.5%; 1 watershed*)	0 (0%)
MCA + PCA	5 (16.1%; 1 watershed*)	1 (2.5%; 1 watershed*)
ACA + MCA + PCA	2 (6.5%)	1 (2.5%)
Multifocal punctate ischemia	1 (3.2%)	2 (5%)
Watershed only	0 (0%)	2 (5%)

N, number; MCA, middle cerebral artery, PCA, posterior cerebral artery, ACA, anterior cerebral artery; watershed* indicates watershed injury in addition to stroke in arterial distribution.

**Figure 3:**
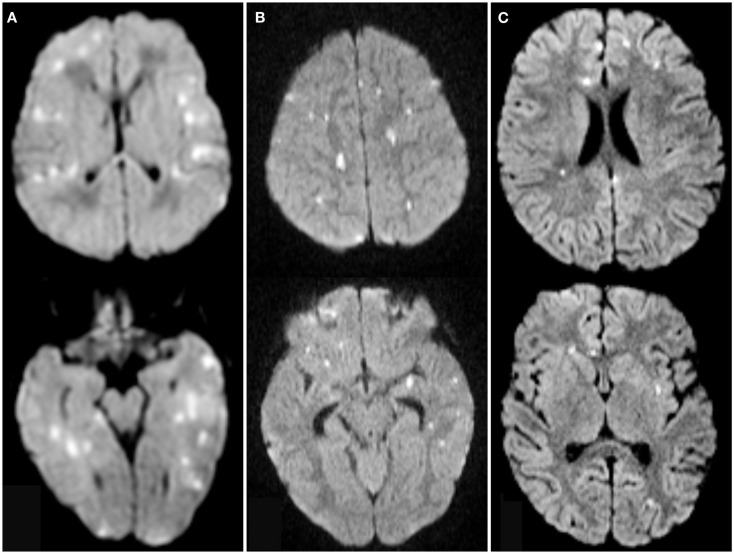
**Axial diffusion weighted images from one neonate (A) and two children (B,C) with multifocal punctate ischemic foci without large or medium vessel stroke demonstrating diffusion restriction in multiple ASPECTS territories**. Scores were 15 **(A)**, 21 **(B)**, and 11 **(C)**.

Two childhood subjects and one perinatal subject had multifocal punctate ischemia without large or medium sized vessel stroke (Figure 3). These three subjects had infarct volume <5% SBV.

### Validity of modified pediatric ASPECTS for assessing stroke volume

The correlation between the modified pediatric ASPECTS and the quantitative infarct volume was strongly positive for the neonates and moderately positive for the childhood subjects when all subjects were included. The correlation was strongly positive in both groups when excluding the subjects with multifocal punctate ischemia without large or medium sized vessel stroke. For the perinatal subjects, the Spearman rank correlation coefficient (ρ) was 0.76, *p* < 0.001. Excluding the single subject with multifocal punctate ischemia, ρ was 0.87, *p* < 0.001. For the childhood subjects, ρ was 0.69, *p* < 0.001. Excluding the two childhood subjects with multifocal punctate ischemia due to meningitis without large or medium sized vessel stroke, ρ was 0.80, *p* < 0.001.

### Interrater reliability of modified pediatric ASPECTS

The median total modified pediatric ASPECTS for perinatal and childhood subjects for the three raters was not significantly different. The median total modified pediatric ASPECTS for the perinatal subgroup for raters 1, 2, and 3 was 6 (IQR 4–10), 6 (IQR 3–12), and 6 (IQR 4–11), respectively (*p* = 0.89, Kruskal–Wallis rank test). The median total modified pediatric ASPECTS for the childhood subgroup for raters 1, 2, and 3 was 4 (IQR 2–8), 4 (IQR 3–7), and 3.5 (IQR 2–6.5), respectively (*p* = 0.66, Kruskal–Wallis rank test). The ICC for the perinatal subgroup was 0.93 (95% confidence interval, CI 0.89–0.97, *p* < 0.001). The ICC for the childhood subgroup was 0.94 (95% CI 0.91–0.97, *p* < 0.001).

### Classification of prognostic group using modified pediatric ASPECTS

In a prior study of children with AIS, infarct volume ≥5% of SBV was predictive of poor outcome and of HT (Beslow et al., 2011). For the current study, an infarct volume ≥5% of SBV was classified as large and an infarct volume <5% of SBV was classified as small. Of the 38 childhood subjects with large or medium vessel stroke, 15 had large infarcts and 23 had small infarcts. A modASPECTS of 5 maximized the sensitivity for differentiating large from small infarcts. Of 114 modified pediatric ASPECTS ratings, 96 (84.2%) correctly classified infarct volume as large or small. In 9 of the 18 discordant ratings, the modified pediatric ASPECTS was ≥5 but the infarct was small. In nine of the discordant ratings, the modified pediatric ASPECTS was <5 but the infarct volume was large. The sensitivity and specificity of a modASPECTS of ≥5 for correctly predicting a large infarct were 80% (95% CI 65.4–90.4%) and 87% (95% CI 76.7–93.9%), respectively. The area under the receiver operating characteristic curve was 0.90 (95% CI 0.84–0.96). Of the 30 perinatal subjects with large or medium vessel stroke, 20 had large infarcts and 10 had small infarcts. Of 90 modified pediatric ASPECTS ratings, 75 (83.3%) correctly classified infarct volume as large or small. In 6 of the 15 discordant ratings, the modified pediatric ASPECTS was ≥5 but the infarct was small. In 9 of the discordant ratings, the modified pediatric ASPECTS was <5 but the infarct volume was large. The sensitivity and specificity of a modASPECTS of ≥5 for correctly predicting a large infarct were 85% (95% CI 73.4–92.9%) and 80% (95% CI 61.4–92.3%), respectively. The area under the receiver operating characteristic curve was 0.83 (95% CI 0.74–0.91).

## Discussion

This study demonstrates that a modified pediatric ASPECTS on acute MRI estimates arterial ischemic infarct volume as a percent of SBV for perinatal and childhood infarction with excellent interrater reliability and validity. The method is attractive since the modified pediatric ASPECTS can be performed in just a few minutes compared to manual segmentation image analysis which takes up to several hours and requires post-processing of the images. Additionally, certain image formats may not be compatible with manual segmentation programs, while the modified pediatrics ASPECTS can be performed on routine clinical viewing stations as long as DWI with ADC is available. The score can also be used to classify infarct volume as large versus small according to the threshold of ≥5% SBV with high sensitivity and specificity. The threshold of 5% of SBV is important for childhood stroke because it predicts both HT of stroke and poorer outcome at follow-up on the Pediatric Stroke Outcome Measure (Beslow et al., 2011).

Our study has several limitations. A region was scored as positive even if a small part of a modified pediatric ASPECTS score region was infarcted. Furthermore, each involved region in the modified pediatric ASPECTS is given equal weight (one point) even though areas like the M1–M6 represent more volume than smaller areas like the internal capsule, insula, or caudate. Both of these factors may cause the modified pediatric ASPECTS to overestimate the infarct volume as a percent of SBV in some patients. An extreme scenario for which the modified pediatric ASPECTS overestimates stroke volume is multifocal punctate ischemia, as demonstrated by the great improvement of the Spearman rank correlation coefficient when excluding such patients (one subject in perinatal group, two subjects in childhood group). Conversely, a subject with stroke affecting fewer than five of the MCA regions could still have a stroke volume ≥slant5% SBV if the entire regions are affected, thereby causing the modified pediatric ASPECTS to underestimate the true stroke volume as a percent of SBV. About 15% of subjects were classified incorrectly as large versus small infarcts, and misclassifications were evenly distributed in either direction. Therefore, one should consider scenarios in which classification could be incorrect when applying the score. Despite these limitations, both the sensitivity and the specificity of the method were high. However, we do not recommend the use of the modified pediatric ASPECTS for use in children with isolated multifocal punctate ischemia. A strength of the study is that the raters had different backgrounds and experience, including radiologists of differing clinical training levels and a pediatric neurologist. Moreover, the raters used two different software packages for viewing the images for the ratings with a high degree of reliability, indicating that modified pediatrics ASPECTS can be performed on a variety of image viewers. Replication of the study involving raters who have other training such as intensivists or research assistants might be useful in order to expand the use of the modified pediatric ASPECTS.

The ease of use of the modified pediatric ASPECTS, along with excellent interrater reliability and validity suggest it could be a useful tool for both researchers and clinicians. For example, use of the modified pediatric ASPECTS could expedite eligibility determination in intervention trials that require stratification by infarct size, such as the planned dose finding and safety study of intravenous recombinant tissue plasminogen activator in pediatric stroke (Amlie-Lefond et al., 2009). The modified pediatric ASPECTS may be a useful tool to help stratify potential subjects according to risk of HT, particularly because many childhood strokes occur in distributions other than that of the middle cerebral artery. However, more research on the modified pediatric ASPECTS in a prospective cohort is necessary to confirm its utility as a predictor of outcome.

## Conclusion

The easily performed modified pediatric ASPECTS on acute MRI represents a useful tool for estimating AIS volume as a percent of SBV in neonates and children with acute AIS. Prospective studies are required to confirm its use for identifying children at risk for HT or poor long term outcome. In the future, this could be a valuable tool for easily and reliably stratifying subjects according to infarct volume for clinical trials and for outcome prediction.

## Conflict of Interest Statement

R. N. Ichord: Consultant/Advisory Board, Berlin Heart Clinical Event Committee.
